# Photonics-Based Simultaneous DFS and AOA Measurement System without Direction Ambiguity

**DOI:** 10.3390/mi14020457

**Published:** 2023-02-15

**Authors:** Qingqing Meng, Zihang Zhu, Guodong Wang, He Li, Lingrui Xie, Shanghong Zhao

**Affiliations:** Information and Navigation College, Air Force Engineering University, Xi’an 710077, China

**Keywords:** doppler frequency shift, angle of arrival, microwave photonics, Sagnac loop

## Abstract

A novel scheme that can simultaneously measure the Doppler frequency shift (DFS) and angle of arrival (AOA) of microwave signals based on a single photonic system is proposed. At the signal receiving unit (SRU), two echo signals and the reference signal are modulated by a Sagnac loop structure and sent to the central station (CS) for processing. At the CS, two low-frequency electrical signals are generated after polarization control and photoelectric conversion. The DFS without direction ambiguity and wide AOA measurement can be real-time acquired by monitoring the frequency and power of the two low-frequency electrical signals. In the simulation, an unambiguous DFS measurement with errors of ±3 × 10^−3^ Hz and a −90° to 90° AOA measurement range with errors of less than ±0.5° are successfully realized simultaneously. It is compact and cost-effective, as well as has enhanced system stability and improved robustness for modern electronic warfare systems.

## 1. Introduction

Accurately judging the condition of a moving object, such as speed, distance and direction, plays a significant role in radar, electronic warfare and communication systems [1,2,3,4]. Owing to the inherent advantages of photonics of large bandwidth, immunity to electromagnetic interference and low transmission loss, various photonic-assisted doppler frequency shift (DFS) and angle of arrival (AOA) measurement techniques to meet the requirement have been reported [5,6,7,8,9,10,11,12]. DFS is the frequency shift between the transmitted microwave signal toward object and the received echo signal returned from an object. It is applied to obtain the velocity and the direction of the moving object [13]. On the other hand, AOA is the azimuth angle of the incoming signal radiation source relative to the receiving antenna, by which the orientation of the measured object can be precisely identified. However, there are still many problems to solve; for example, the direction of DFS is difficult to judge, and the problem of ambiguity in the AOA. Moreover, most of the previously reported systems can only measure either DFS or AOA, which limits the application of the systems and hard to fast obtain the accurate location of the subject.

In recent years, some photonic schemes aimed at simultaneously implementing DFS and AOA measurements based on a single system are being studied. In 2019, Peng Li et al. [14] first proposed a photonic approach for simultaneously measuring the DFS and the AOA by connecting two modulators, polarization-division-multiplexed Mach–Zehnder modulator (PDM-MZM) and phase modulator (PM), in series. It can not only realize the DFS measurement error of less than 5 × 10^−3^ Hz, and the AOA from 1.82° to 90° with less than 0.85° error at 10 GHz and from 4.35° to 90° with less than 2.25° at 18 GHz, but also support long-distance transmission. During the same year, an impressive scheme that both parameters can be measured simultaneously by constructing two in-phase and quadrature (I/Q) channels is provided in [15]. The DFS measurement error is less than 2.5 Hz and the AOA error is less than 2.3° between 27.25° and 90°. Nevertheless, both systems are complicated. In 2020, a cascaded modulator topology for radio frequency (RF) signal direction finding is presented in [16], which can simultaneously measure the two parameters of an RF signal when it is used in a radar receiver. It can perform the DFS measurement error less than 0.8 Hz and the AOA measurement error less than ±2.5° in a range from 3.2° to 81.5°, and it can be extended to a multi-channel system. In 2021, two simple photonics-based DFS and AOA estimation systems based on a polarization-division-multiplexed dual-drive Mach–Zehnder modulator (PDM-DMZM) [17] or a dual-parallel Mach–Zehnder modulator (DPMZM) [18] are implemented. Especially in [18], the phase ambiguity problem is solved and the AOA measurement range is from ±10° to ±90° with a measurement error of less than 5.5°. Whereas, an optical bandpass filter (OBPF) or a wavelength-division multiplexer (WDM) is required to select or separate the optical signals, restricting the operating range of the whole system. In 2022, a photonic approach for simultaneously measuring DFS and AOA based on a dual-polarization dual-drive Mach–Zehnder modulator (DPol-DDMZM) is proposed [19], which can not only measure DFS without detection ambiguity, but also acquire the unambiguous AOA measurement with a range from −66.44° to 66.44° and errors of less than ±2.2°. However, a power coupler must combine the received echo signal and the reference signal before driving the modulator. It is difficult to achieve synchronization in practical applications, which will affect measurement accuracy.

In electronic warfare, it is necessary to transmit high-power signals to measure the characteristics of enemy targets, which will increase the possibility of our system being found by the enemy. Once being discovered, it is likely to encounter interference or deception from the enemy, or even the attack to destroy the entire measurement system. Under this circumstance, we propose and demonstrate a photonics-based simultaneous DFS and AOA measurement system with the capability of unambiguity detection. To improve the concealment and security of the central station (CS), the microwave signal receiving unit (SRU) and the CS are separated and far away from each other, which has hardly been considered in the aforementioned methods. In the remote SRU, only one Sagnac loop is employed to receive and modulate signals, resulting in an improved robustness against the environmental perturbations. Then, the modulated optical signal is transferred to the signal processing unit (SPU) in the CS by a segment of single mode fiber (SMF). The DFS (including values and direction) and the non-ambiguous AOA in the range of 180° can be realized by measuring the frequency and power of the output signals in the CS. The advantages and novelties of our scheme are that a single photonic system can simultaneously realize an unambiguous DFS measurement with errors of ±3 × 10^−3^ Hz and a −90° to 90° AOA measurement range with errors of less than ±0.5°. Most of aforementioned work can only realize AOA with a measurement range of no more than 90°, e.g., refs. [12,14,15,16], which restricts the real-world application. To improve the concealment and security of the central station (CS), the microwave signal receiving unit (SRU) and the CS are separated and far away from each other, although it was mentioned in [14], its system structure is relatively complex and the analysis of the influence of optical fiber transmission on signals is rough. In addition, in our scheme, only one Sagnac loop is employed to receive and modulate signals in the remote SRU, resulting in an improved robustness against the environmental perturbations. In addition, the filtering elements in optical domain such as optical filter or WDM are not necessary in our design. It is compact and cost-effective, as well as has enhanced system stability and improved robustness for modern electronic warfare systems.

## 2. Principle

**Figure 1:**
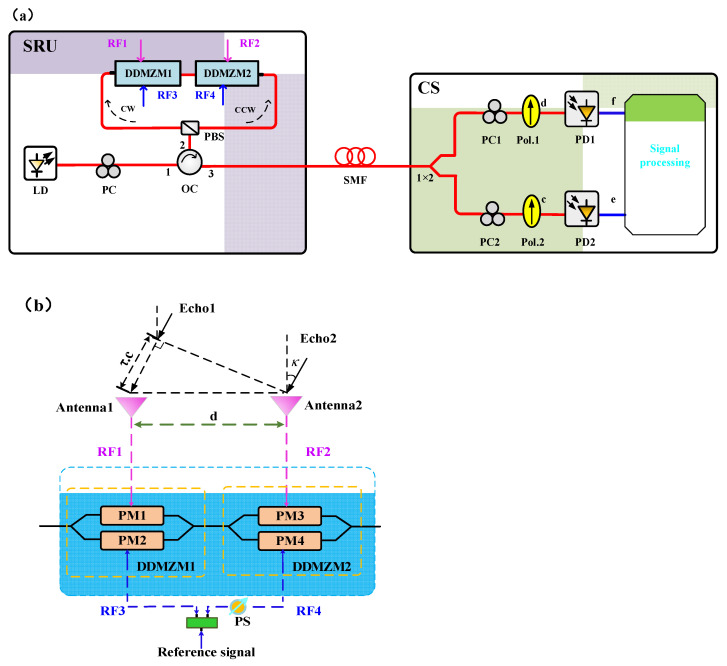
Schematic diagram of (**a**) the system for simultaneous DFS and AOA measurement. LD: laser diode; PC: polarization controller; OC: optical circulator; PBS: polarization beam splitter; DDMZM: dual-drive Mach–Zehnder modulator; SMF: single mode fiber; Pol: polarizer; PD: photodetector; PM: phase modulator; PS: phase shifter; CS: the central station, SRU: signal receiving unit. (**b**) Configuration of DDMZM1 and DDMZM2.

The schematic diagram of the proposed approach for the simultaneous and unambiguous measurement of the DFS and AOA is shown in Figure 1a. With a certain length of single mode fiber, remote measurement of AOA and DFS can be realized. In the remote SRU, an optical carrier from a laser diode (LD) is coupled into the Sagnac loop via a polarization controller (PC). When the linearly polarized light is adjusted by the PC, two orthogonally polarized signals (X-polarized and Y-polarized) are generated and injected into port1 of an optical circulator (OC) and outputs from port2 that connects to the Sagnac loop. In the Sagnac loop, two orthogonally polarized signals are divided by the PBS so that one part propagates along the clockwise (CW) direction and the other part propagates along the counterclockwise (CCW) direction. In addition, the optical signal propagating in the CW direction is only effectively modulated in a dual-drive Mach–Zehnder modulator (DDMZM1), while the optical signal propagating in the CCW direction is only effectively modulated in a dual-drive Mach–Zehnder modulator (DDMZM2) [20]. Subsequently, the the X-polarized and Y-polarized modulated light waves are combined into a polarized multiplexed signal by the PBS and output from port3 of OC, and then transmitted to the CS through the SMF. In the CS, the optical signal is separated into two beams by a 1 × 2 optical power splitter. In the upper and lower branch, the polarized multiplexed signal is combined into a linearly polarized lightwave by a polarization controller (PC) and polarizer (Pol) and then detected by the photodetector (PD), respectively.

At the remote SRU, both DDMZM consist of two parallel-connected PMs in a main MZI, with two RF input ports and a DC port, respectively. A reference microwave signal is divided into two identical microwave signals (RF3 and RF4) by a power coupler. The echo signals received by two antennas and the reference signals are, respectively, sent to the two input RF ports of DDMZM1 and DDMZM2, as can be seen in Figure 1b. DDMZM1 and DDMZM2 are both biased at maximum point. The output signal of the DDMZM1 and the DDMZM2 can be expressed as:

(1) $$E_{in}= [\begin{array}{l} E_{x}(t)\\ E_{y}(t) \end{array}]\propto E_{0}exp(j\omega_{c}t) [\begin{array}{l} e^{jm_{e}sin(\omega_{e}t+\theta )}+e^{jm_{r}sin(\omega_{r}t+\varphi )}\\ e^{jm_{e}sin(\omega_{e}t)}+e^{jm_{r}sin(\omega_{r}t)} \end{array}]$$

where *E_x_*(*t*) and *E_y_*(*t*) are the optical fields of the CW and CCW, *E*_0_ and *ω_c_* are the amplitude and angular frequency of the input optical signal, *ω_e_* and *ω_r_* are the angular frequency of the echo signal and reference signal, respectively, *m_e_* = π*V*_1_/*V_π_* and *m_r_* = π*V*_2_/*V_π_* are modulation indices for the two echo signals and reference signal, *V_π_* denotes half-wave voltage of the DDMZM1 and DDMZM2, *V*_1_ and *V*_2_ are the amplitude of the two echo signals and reference signal, respectively. *φ* is the phase difference between the two reference signals, which is introduced by a phase shifter. *θ* the phase difference of the two echo signals, which is introduced by the AOA *κ* and antenna spacing *d* (usually *λ*/2). The AOA can be expressed by the phase difference as [19].

(2) $$\kappa =arcsin(\frac{\theta }{\pi })$$

Afterwards, the polarized multiplexed signal is transmitted to the CS by the SMF, which would introduce a phase shift to the optical signal. In the CS, the polarized multiplexed signal is equally split into the upper and lower paths through a 1 × 2 optical power splitter. By turning the state of the cascaded PC and the position of the Pol, the output lightwave can be expressed as

(3) $$E_{out}(t)=cos(\alpha )E_{x}(t)+sin(\alpha )E_{y}(t)e^{j\psi_{PC}}$$

where α is the polarization angle between the axial direction of the polarizer and the principal axis, and $\psi_{PC}$ is the phase shift between the two orthogonal polarization states introduced by PC. Under the small-signal condition, the optical signals along the upper branch and the lower branch can be concluded as:

(4) $$\begin{array}{c} E_{out}(t)\propto cos(\alpha )E_{in}e^{j\omega_{c}t}\left[\begin{array}{l} J_{0}(m_{e})e^{j\varphi_{0}}+J_{1}(m_{e})e^{j\omega_{e}t+j\theta +j\varphi_{-1}}-J_{1}(m_{e})e^{-j\omega_{e}t-j\theta +j\varphi_{-1}}\\ +J_{0}(m_{r})e^{j\varphi_{0}}+J_{1}(m_{r})e^{j\omega_{r}t+j\varphi +j\phi_{-1}}-J_{1}(m_{r})e^{-j\omega_{r}t-j\varphi +j\phi_{-1}} \end{array}\right]\\ +sin(\alpha )E_{in}e^{j\omega_{c}t}\left[\begin{array}{l} J_{0}(m_{e})e^{j\varphi_{0}}+J_{1}(m_{e})e^{j\omega_{e}t+j\varphi_{-1}}-J_{1}(m_{e})e^{-j\omega_{e}t+j\varphi_{-1}}\\ +J_{0}(m_{r})e^{j\varphi_{0}}+J_{1}(m_{r})e^{j\omega_{r}t+j\phi_{-1}}-J_{1}(m_{r})e^{-j\omega_{r}t+j\phi_{-1}} \end{array}\right]e^{j\psi_{PC}} \end{array}$$

where *J_n_* (·) denotes the *n*th-order Bessel function of the first kind. The additional phase of different frequency components caused by the fiber chromatic dispersion can be expressed as:

(5a) $$\delta_{0}=L\beta_{0}\left(\omega_{c}\right)$$

(5b) $$\delta_{-1}=L\beta \left(\omega_{c}-\omega_{n}\right)\approx L\beta_{0}\left(\omega_{c}\right)-L\beta_{1}\left(\omega_{c}\right)\omega_{n}+\frac{1}{2}L\beta_{2}\left(\omega_{c}\right)\omega_{n}^{2}$$

(5c) $$\delta_{+1}=L\beta \left(\omega_{c}+\omega_{n}\right)\approx L\beta_{0}\left(\omega_{c}\right)+L\beta_{1}\left(\omega_{c}\right)\omega_{n}+\frac{1}{2}L\beta_{2}\left(\omega_{c}\right)\omega_{n}^{2}$$

$\delta_{0}$, $\delta_{-1}$, and $\delta_{+1}$ are the phase shifts caused by the fiber chromatic dispersion on the optical carrier at *ω_c_*, the lower sideband at *ω_c_* − *ω_n_* and the upper sideband at *ω_c_ + ω_n_*, respectively. *β_n_* (*n* = 0, 1, 2) is the *n*th-order dispersion coefficient, *L* is the length of SMF. Based on Equation (5a–c), $\varphi_{0}=\delta_{0}$; $\varphi_{+1}=\delta_{+1}$ and $\varphi_{-1}=\delta_{-1}$ as *ω_n_* = *ω_e_*; $\phi_{+1}=\delta_{+1}$ and $\phi_{-1}=\delta_{-1}$ as *ω_n_* = *ω_r_*.

To achieve the unambiguous AOA measurement with a range of 180°, different value of α and $\psi_{PC}$ will be introduced to the upper and lower branch. When tuning $\alpha_{1}=45^{{}^{\circ}}$, $\psi_{PC1}=0^{{}^{\circ}}$ and $\alpha_{2}=135^{{}^{\circ}}$, $\psi_{PC2}=90^{{}^{\circ}}$, thence, the output optical field of the upper and lower branch can be written as:

(6) $$\left\{ \begin{array}{l} E_{out1}(t)\propto E_{in}e^{j\omega_{c}t}\left[\begin{array}{l} 2J_{0}(m_{e})e^{j\varphi_{0}}+J_{1}(m_{e})e^{j\omega_{e}t+j\varphi_{+1}}(1+e^{j\theta })-J_{1}(m_{e})e^{-j\omega_{e}t+j\varphi_{-1}}(1+e^{-j\theta })\\ +2J_{0}(m_{r})e^{j\varphi_{0}}+J_{1}(m_{r})e^{j\omega_{r}t+j\phi_{+1}}(1+e^{j\varphi })-J_{1}(m_{r})e^{-j\omega_{r}t+j\phi_{-1}}(1+e^{-j\varphi }) \end{array}\right]\\ E_{out2}(t)\propto E_{in}e^{j\omega_{c}t}\left[\begin{array}{l} J_{1}(m_{e})e^{j\omega_{e}t+j\varphi_{+1}}(e^{j\theta }-e^{j\frac{\pi }{2}})-J_{1}(m_{e})e^{-j\omega_{e}t+j\varphi_{-1}}(e^{-j\theta }-e^{j\frac{\pi }{2}})\\ +J_{1}(m_{r})e^{j\omega_{r}t+j\phi_{+1}}(e^{j\varphi }-e^{j\frac{\pi }{2}})-J_{1}(m_{r})e^{-j\omega_{r}t+j\phi_{-1}}(e^{-j\varphi }-e^{j\frac{\pi }{2}}) \end{array}\right] \end{array}\right.$$

The value of DFS is the frequency offset of the echo signal and the transmitted signal, i.e., $f_{DFS}=f_{e}-f_{t}$. In most real-world application, the DFS value is between −1 MHz and 1 MHz [14], so the frequency of the reference signal is set as the frequency of the transmitted signal plus 2 MHz, that is, $f_{r}=f_{t}+2\;\mathrm{MHz}$. The value of DFS can be expressed $f_{DFS}=f_{e}-f_{r}+2\;\mathrm{MHz}=-(f_{r}-f_{e})+2\;\mathrm{MHz}$, which means the DFS can be obtained by processing the received intermediate frequency (IF) electrical signals. The photocurrents at the angular frequency of $\Delta w=\left|w_{e}-w_{r}\right|$ can be obtained from Equation (6) and expressed by:

(7) $$\left\{\begin{array}{ll} I_{upper} & \propto P_{in}J_{1}(m_{e})J_{1}(m_{r})cos(\frac{\theta }{2})cos(\frac{\varphi }{2})cos(\frac{\left(\varphi_{+1}+\varphi_{-1})-(\phi_{+1}+\phi_{-1}\right)}{2})\\ & cos((\omega_{e}-\omega_{r})t+\frac{\theta -\varphi +(\varphi_{+1}-\varphi_{-1})-(\phi_{+1}-\phi_{-1})}{2})\\ I_{lower} & \propto P_{in}J_{1}(m_{e})J_{1}(m_{r})\sqrt{A^{2}+B^{2}}\\ & cos((\omega_{e}-\omega_{r})t+\frac{\theta -\varphi +(\varphi_{+1}-\varphi_{-1})-(\phi_{+1}-\phi_{-1})}{2}-arctan\frac{B}{A}) \end{array}\right.$$

where $A=cos\frac{\left(\varphi_{+1}+\varphi_{-1})-(\phi_{+1}+\phi_{-1}\right)}{2}cos(\frac{\theta -\varphi }{2})$, $B=sin\frac{\left(\varphi_{+1}+\varphi_{-1})-(\phi_{+1}+\phi_{-1}\right)}{2}sin(\frac{\theta +\varphi }{2})$. The frequency of the photocurrent is between 1 MHz and 3 MHz. Therefore, the value of DFS and the moving direction of the target can be determined, which corresponds to frequency of low-frequency electrical signal ranges from 1 to 2 MHz (positive) and 2 to 3 MHz (negative). Consequently, the DFS can be easily estimated through the spectrum analysis by a low-frequency ESA.

The powers of the two paths are as follows:

(8) $$\left\{\begin{array}{l} P_{upper}\propto P_{in}^{2}J_{{}_{1}}^{2}(\omega_{e})J_{{}_{1}}^{2}(\omega_{r})(1+cos(\theta ))(1+cos(\varphi ))(1+cos((\varphi_{+1}+\varphi_{-1})-(\phi_{+1}+\phi_{-1})))\\ P_{lower}\propto P_{in}^{2}J_{{}_{1}}^{2}(\omega_{e})J_{{}_{1}}^{2}(\omega_{r})(1+sin(\theta )sin(\varphi )+cos(\theta )cos(\varphi )cos((\varphi_{+1}+\varphi_{-1})-(\phi_{+1}+\phi_{-1}))) \end{array}\right.\;$$

**Figure 2:**
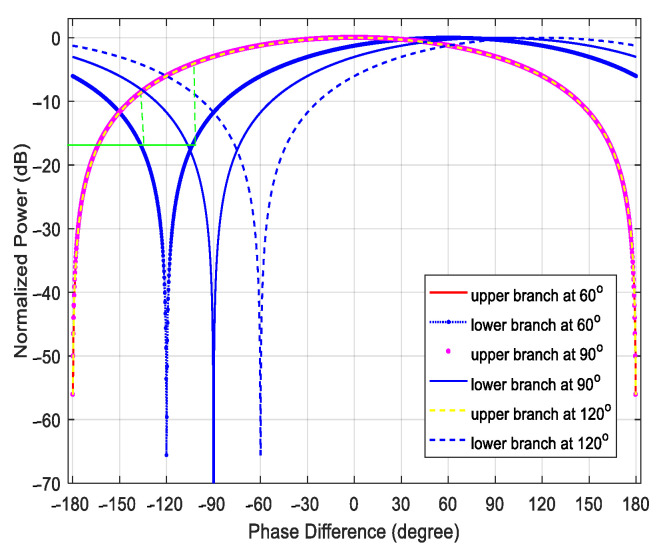
The normalized power of the output signals versus phase difference of the two echo signals at different phase difference between the two reference signals.

Two mapping curves on the relationship between the phase difference and the power of microwave signals are constructed when the parameters of the SMF and the phase difference between the two reference signals *φ* are fixed, which can be seen from Figure 2. Here, choosing the lower branch as the phase measurement curve and the upper branch as the ambiguity resolution curve, although the same power corresponds to two phase values in the blue power-phase mapping curve, the two phase values correspond to different powers in the red line, the system can realize the unambiguous phase measurement in a range of 360°, as the green line shows. The unambiguous AOA measurement with a range of −90° to 90° can be further calculated. Meanwhile, it is noticed from Figure 2 that the phase difference between the two reference signals *φ* can control the distance between these two mapping curves, and with the decrease in *φ*, the power difference is larger, which means we can obtain an accurate measurement result of phase difference.

## 3. Analysis and Simulation Results

To investigate its mechanism, simulations based on the structure shown in Figure 1 are performed via the OptiSystem 15.0 (Optiwave Systems, Ontario, Canada). The CW laser diode emits the lightwave with the central wavelength of 1550 nm, line width of 1 MHz and power of 12 dBm. The polarization state of the incident lightwave is aligned with an angle of 45° by PC. It is transmitted over the Sagnac loop via a circulator. The transmitted signal is set as 15 GHz and a 2.0 a.u. sinusoidal signal with the frequency 15.002 GHz is used as a reference signal, which is divided into two microwave signals (RF3 and RF4). To simplify the formula, a constant phase difference between RF3 and RF4 is introduced by a 90° hybrid coupler. The other sinusoidal signal is split into two identical echo signals, and an additional phase shift is introduced into one of echo signals by a phase shifter. They drive two DDMZMs as described in the principal part. Both DDMZMs are controlled at the maximum point and have a half-wave voltage of 3.5 V at each electrode. A spool of 5km SMF with dispersion coefficient of 17 ps/nm/km is used to connect the SRU and the CS. The device angle of linear polarizer is tuned at 45° in the upper branch. In the lower branch, the device angle of linear polarizer is tuned at 135° and the phase shift of polarization phase shift is 90°. Two PDs with the same 1 A/W responsivity and 10 nA dark current are used to convert optical signals back to electrical signals, and two electrical spectrum analyzers (ESA) are used to observe the frequency and the power of the IF signals.

For the case with a fixed phase shift of reference signals of *φ* = 90°, Equation (8) can be simplified as:

(9) $$\left\{\begin{array}{l} P_{upper}\propto P_{in}^{2}J_{{}_{1}}^{2}(\beta_{e})J_{{}_{1}}^{2}(\beta_{r})(1+cos(\theta ))(1+cos((\varphi_{+1}+\varphi_{-1})-(\phi_{+1}+\phi_{-1})))\\ P_{lower}\propto P_{in}^{2}J_{{}_{1}}^{2}(\beta_{e})J_{{}_{1}}^{2}(\beta_{r})(1+sin(\theta )) \end{array}\right.$$

According to Equation (5a–c) and the second-order dispersion coefficient $\beta_{2}^{}=-\lambda^{2}D/2\pi c$, Equation (9) can be rewritten as:

(10) $$\left\{\begin{array}{l} P_{upper}\propto P_{in}^{2}J_{{}_{1}}^{2}(\beta_{e})J_{{}_{1}}^{2}(\beta_{r})(1+cos(\theta ))(1+cos(\lambda^{2}DL(\omega_{e}^{2}-\omega_{r}^{2})/4\pi c))\\ P_{lower}\propto P_{in}^{2}J_{{}_{1}}^{2}(\beta_{e})J_{{}_{1}}^{2}(\beta_{r})(1+sin(\theta )) \end{array}\right.$$

where *D* and *L* represent the dispersion parameter and length of the SMF, respectively. *λ* denotes the wavelength of the optical carrier and *c* is the velocity of light in vacuum. From Equation (10), the power of the lower branch is independent to the phase shift caused by the fiber chromatic dispersion. Therefore, choosing the lower branch as the phase measurement curve and the upper branch as the ambiguity resolution curve is a better choice.

**Figure 3:**
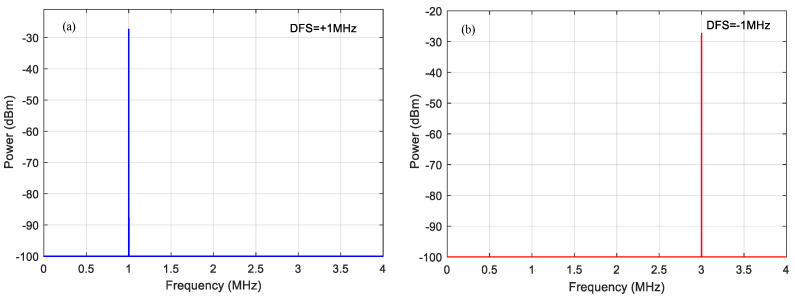
Electrical spectra in the lower branch for the Doppler frequency shifts at (**a**) +1 MHz and at (**b**) −1 MHz.

When the echo microwave signal frequency is set to 15.001 GHz with the amplitude of 1.8 a.u., a peak with a frequency of 1 MHz and a power of −27.6 dBm displays on the ESA, as shown in Figure 3a. It can be seen that the simulated values agree well with the theoretical ones. The value of this frequency means the DFS is +1 MHz and the direction of DFS is positive and one target is moving towards the receiver. Changing the echo signal from 15.001 GHz to 14.999 GHz, the frequency of the peak is located at 3 MHz in Figure 3b, which illustrates that the value of DFS is −1 MHz and a target is moving away from the receiver. Compared with the measurement schemes in refs. [14,15,17,18], they need to display the waveforms of the two channels on the oscilloscope (OSC), and compare the phase relationship between the two channels, thereby the proposed scheme is simpler and more feasible.

**Figure 4:**
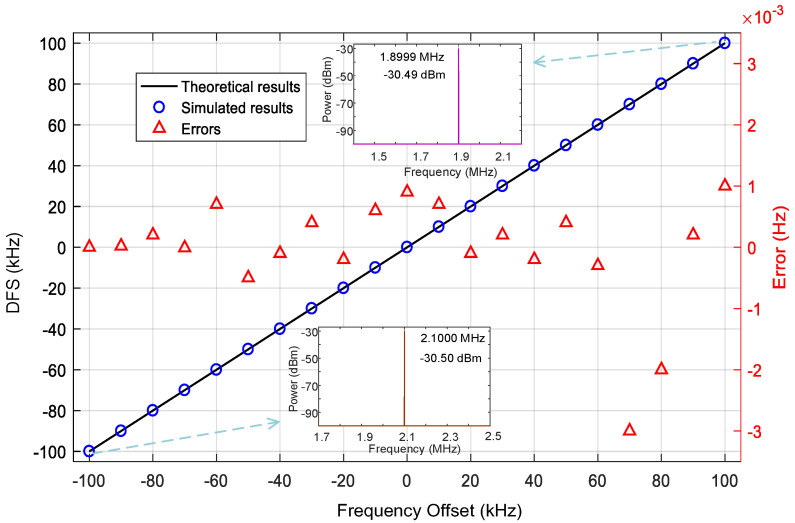
The simulated (dot) and the actual DFS (solid line) results from −100 kHz to 100 kHz with a step of 10 kHz and the corresponding errors (red triangles). To clearly show the simulated results, the electrical spectra at the DFSs of ±100 kHz are also shown in the insets of Figure 4 respectively.

Next, a series of different echo signals are sited to measure DFS and analyze errors. The phase difference between two echo signals is fixed at 0° and the reference signal frequency is fixed at 15.002 GHz while the frequencies of echo signals change from 15 GHz −100 kHz to 15 GHz +100 kHz at intervals of 10 kHz. Simulated results of each DFS are obtained from the frequency of IF signal and the measurement errors can be calculated by comparing the values of the measured DFS and practical frequency offsets, as shown in Figure 4. It can be seen that the simulated values of DFS and the theoretical values agree well with each other, and the maximum DFS measurement error is within ±3 × 10^−3^ Hz.

**Figure 5:**
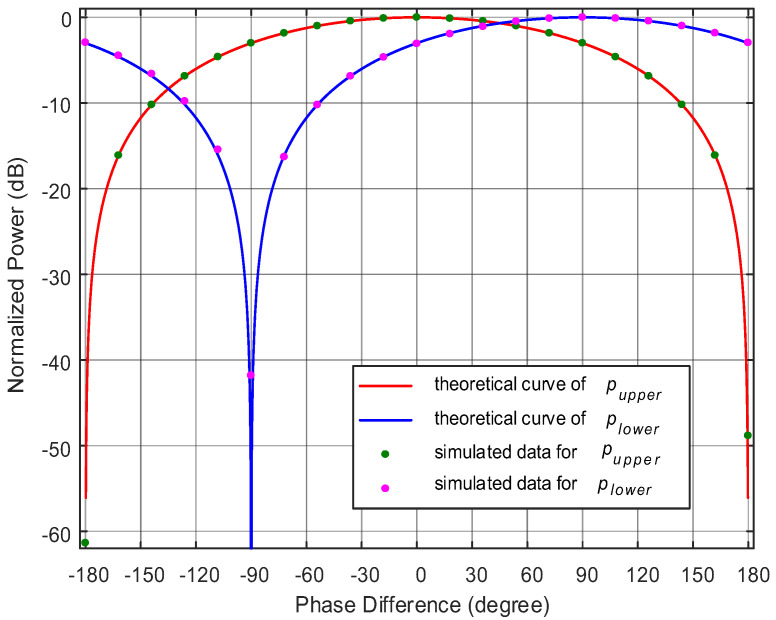
The simulated results of actual data and theoretical curve for normalized power of the output signals versus phase difference of the two echo signals.

Subsequently, the echo microwave signal frequency is fixed at 15.001 GHz while a variable phase difference between the two echo signals is adjusted by controlling the phase shifter, and 21 data points are measured in the range from −180° to 180°. The normalized theoretical curve obtained from Equation (10) and measured normalized power of the output electrical signal versus phase difference of the two echo signals are shown in Figure 5. As can be seen, the simulated results match well to the theoretical results except from the notch portion. The reason for a big gap around the notches is that the normalized power is infinitesimal when the power is zero in the theoretical formula, which will cause a very small measurement error to the actual measurement for the slope of the curve is pretty stiff. It is also noted from Figure 5 that the phase difference in the range from −180° to 180° can be accurately estimated from these two curves, for one power value maps two same phase values from the lower branch (blue curve), but it corresponds to two different phase values in the upper branch (red curve). This means the system can realize the unambiguous phase measurement within the 360° phase range.

**Figure 6:**
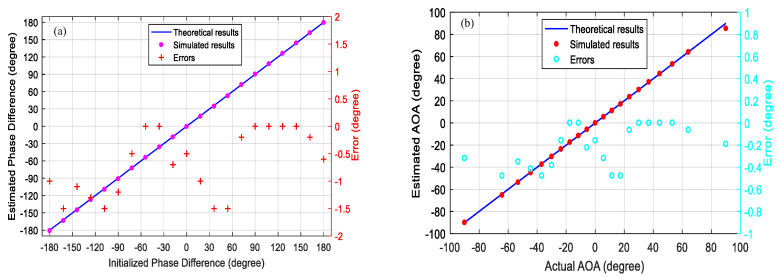
(**a**) Estimated phase difference (rose, dots) and the corresponding error (red, crosses) versus the ideal curve of initialized phase difference from −180° to 180°. (**b**) Estimated AOA (red, dots) and the corresponding error (cyan, circles) versus the ideal curve of actual AOA from −90° to 90°.

Figure 6a demonstrates the ideal curve of initialized phase difference and estimated phase differences from −180° to 180° based on measured normalized power of the output electrical signal and Equation (10), and corresponding measurement errors. As can be seen, the phase differences measurement errors are less than ±1.5° with the range from −180° to 180°. According to the relationship between the AOA and the phase difference given by Equation (2), the simulated results for the actual AOA and the estimated AOA are shown in Figure 6b. Finally, the measurement errors of AOA are less than ±0.5° ranging from −90° to 90°.

**Figure 7:**
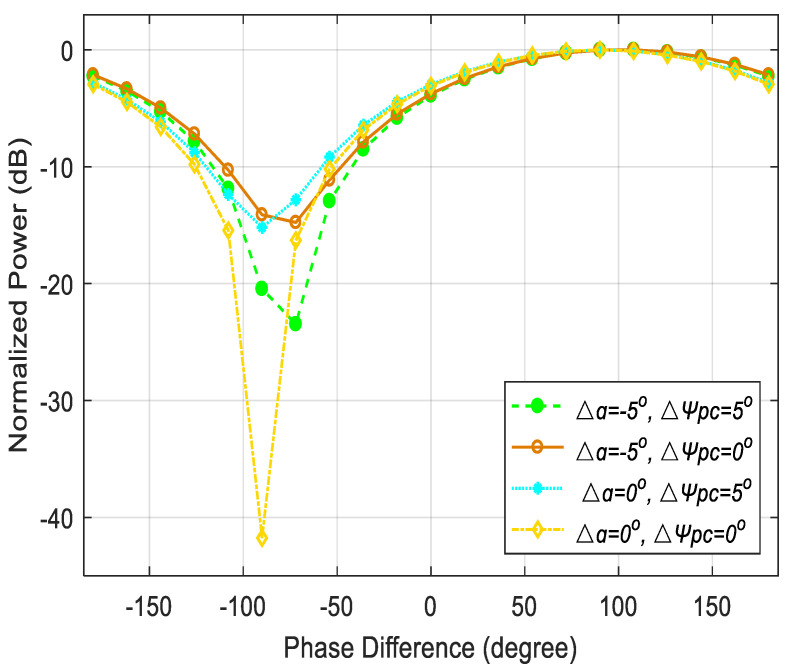
The influence of the polarization angle α and the phase shift $\psi_{PC}$ introduced by PC on normalized power of the output signals.

Some factors that may cause measurement errors such as the direct current (DC) drifting of DDMZMs, phase imbalance of 90° hybrid coupler, the polarization angle and the phase shift introduced by PC drift are also considered and analyzed. Firstly, the DC bias voltage of DDMZMs may drift and affect the performance of the proposed system. Fortunately, even though it will lead to an uncertainty phase shift to *I*_upper_ and *I*_lower_, the direction of the DFS is judged by comparing the frequencies rather than the phases of the output waveform, which avoids the influence of DC drifting. In the simulation, the two reference signals (RF3 and RF4) have a fixed phase shift of 90° by a 90° hybrid coupler. Due to the component imperfection, the instability of the phase shift will introduce the measurement error. To simplify the discussion, when different phase shifts in Figure 2 are considered as phase migration, it is found that phase migration causes the lower output curve to shift left or right, which will bring measurement errors to phase difference measurement. In addition, the polarization angle α and the phase shift $\psi_{PC}$ introduced by PC may drift due to limited device’s accuracy and environment perturbation. Figure 7 shows the measurement results of the lower branch under different polarization states deviation. Apparently, the non-aligned polarization states affect the measurement resolution. According to the measured power value and the relationship between phase difference and AOA, the AOA measurement error is more than ±5° within the angular range from −36.9° to −23.6° when the polarization angle deviation Δα =−5° and the phase shift deviation $\Delta \psi_{PC\;}=5{}^{\circ}$. To enhance the measurement performance, the polarization instability can be resolved by using polarization maintaining components and feedback control. In addition, the potential photonic integration can significantly improve the performance of the structure, making it more suitable for radar and warfare applications.

## 4. Conclusions

In conclusion, we have proposed and analyzed a novel scheme that can simultaneously measure DFS and AOA without detection ambiguity. In this system, the SRU and the CS is separated to ensure the concealment and safety of the CS. At the SRU, a Sagnac loop containing two DDMZMs is constructed to improve its robustness to environmental disturbances. At the CS, two low-frequency electrical signals are received, and two mapping curves are acquired by processing the power of them. The value and the direction of the DFS can be obtained by measuring the frequency of low-frequency signal with errors of less than ±3 × 10^−3^ Hz. Furthermore, by choosing the lower branch as the phase measurement curve and the upper branch as the ambiguity resolution curve, the unambiguous AOA measurement with a range of −90° to 90° and errors of less than ±0.5° is realized. The scheme is compact and cost-effective, as well as enhanced system stability and improved robustness.

## Data Availability

Not applicable.
